# Targeting high-risk pediatric solid tumors with CAR T cells directed against ALK (anaplastic lymphoma kinase, CD246)

**DOI:** 10.1186/2051-1426-2-S3-P40

**Published:** 2014-11-06

**Authors:** Alec Walker, Paola Lopomo, William Babbitt, Marc Vigny, Crystal Mackall, Rimas Orentas

**Affiliations:** 1 Pediatric Oncology Branch, CCR, NCI, NIH, Bethesda, MD, USA, United States; 2 Institut du Fer à Moulin, INSERM/UPMC, Paris, France, France

## 

Neuroblastoma is the most common non-CNS tumor in children. Poor prognosis for patients presenting with high-risk disease underlines the need for novel therapeutic strategies. Chimeric antigen receptor T cell (CART) therapies combine the epitope specificity of a monoclonal antibody with the cytotoxicity of an activated T cell, and have demonstrated clinical efficacy in pediatric leukemia. To expand this approach to neuroblastoma, we focused on anaplastic lymphoma kinase (ALK), which can exist in native, over-expressed, or mutated forms and may help drive disease progression. Single chain variable fragments (scFvs) derived from four different monoclonal antibodies against ALK were used to construct second-generation CAR vectors featuring either CD28/CD3-zeta or CD137/CD3-zeta signaling domains. Some CAR constructs also included an IgG1 CH2CH3 spacer domain between the scFv and transmembrane domain. Retroviral vectors expressing ALK-specific CARs were generated and used to transduce human peripheral blood mononuclear cells. Transduction efficiencies ranged from 8% to 99%, depending on specific characteristics of each CAR construct. The resulting CART lysed ALK-positive neuroblastoma and Ewing's sarcoma cell lines and had minimal activity against an ALK-negative control. The ability to lyse tumor cells varied across scFvs, and short CART (no Ig spacer) showed enhanced lysis compared to long CARs (with Ig spacer). Short CART also produced Th1 cytokines (including IFN-gamma, IL-2, and TNF-alpha) when co-incubated with ALK-positive tumor lines; however, long CARs did not. Additionally, short anti-ALK CART significantly delayed the growth of human neuroblastoma in an NSG xenograft model when tumor-bearing mice were preconditioned with cyclophosphamide. Ongoing studies aim to: 1) further assess anti-ALK CART therapy *in vivo *in combination with modulation of suppressive murine myeloid populations; 2) explore mechanisms of ALK-positive neuroblastoma resistance to CART-mediated eradication; 3) characterize ALK protein expression in murine central nervous system tissues to better understand possibility of on-target, off-tumor toxicities with this therapy. Ultimately, anti-ALK CART could provide a novel therapy for pediatric solid tumors expressing cell-surface ALK.

